# Applying health, safety, and environmental risk assessment at academic settings

**DOI:** 10.1186/s12889-020-09419-5

**Published:** 2020-09-01

**Authors:** Alireza Dehdashti, Farin Fatemi, Muhammadreza Jannati, Fatemeh Asadi, Marzieh Belji Kangarloo

**Affiliations:** 1 Social Determinant of Health Research Center, Semnan University of Medical Sciences, Semnan, Iran; 2 Research center for health sciences and technologies, Semnan University of Medical Sciences, Semnan, Iran; 3 Student Research Committee, Semnan University of Medical Siences, Semnan, Iran

**Keywords:** Risk assessment, Accident, Academic institutions

## Abstract

**Background:**

Students, staff, and faculties are involved in activities that exposed them to a range of minor to severe or even fatal accidents in academic settings. Managing work environment risks is crucial to any safety and health prevention program. This study developed a risk assessment using combinations of hazards and risk factors to establish a scale of measures in a risk reduction action plan.

**Methods:**

This cross-sectional study was conducted in an Iranian medical sciences university in 2018. A structured method of risk assessment was developed, applying a three-step procedure to identify hazards, consequences, and risk evaluation. Data were collected through detailed health, safety, and environment checklist in 38 different sites. Finally, the risks quantified, prioritized, and control measures proposed accordingly. Chi-square and correlation tests assessed how environmental factors were associated with hazard consequences. The analysis results were evaluated at the significance level of 0.05.

**Results:**

The frequencies of moderate and high-risk levels were 22.7 and 2.9%, respectively. Thus, corrective measures should be considered as soon as possible and immediately for these risk groups. Facilities and functions within laboratories, library, and powerhouse were more vulnerable to serious risks. The type of hazard had associated with the sites and total risk score at the significance level of 0.05 (*P*-value = 0.017). Similarly, risk severity was significantly related to the sites (*P*-value = 0.003). Safety hazards had a statistically higher contribution to the total risk score when compared to health and environmental hazards.

**Conclusion:**

The study revealed complex risks and hazardous circumstances with significant variances in academic sites and activities. Universities should provide training in risk reduction programs to increase the awareness of students, staff, and faculties, which can improve life safety in a university environment.

## Background

During the last two decades, universities and academic institutions across Iran have experienced such tragedies that demonstrated their vulnerability against hazards and incidents. The number of accidents has been increased in academic institutions around the world [1]. Accidents may result in damage to equipment and facilities, minor or major injuries, or death [2]. A survey on reported accidents in Iranian higher education institutions showed 60 deaths and more injuries during educational and research activities [3, 4]. Universities are places where young people prepare themselves to work as professionals in different areas. Working activities in academic sites, such as laboratory, may be accompanied by a variety of hazardous risks. While students, staff, and faculties need to stay alert and aware at all times to avoid accidents, managers need to know the most common causes for university accidents and be able to identify in advance the risk factors to prevent them. The integration of Health, Safety, and Environmental (HSE) program has revealed its advantage in managing risk assessment, aiming at preventing accidents and injuries in the work environment [5]. Academic institutions have a primary duty to provide safety and health for the students, staff, and faculties. A risk assessment provides details of any risks linked with facilities or activities, which can then be used in taking preventive actions [6–9]. The literature reviews indicated that the loss of accidents could be mitigated through an effective risk management program. Prior experiences have shown that risk assessment may provide an effective way to handle the probable incidents by applying an appropriate risk assessment [8, 9]. It has been reported that managing HSE risks helped to avoid costly accidents, losses, and injuries in academic settings. Furthermore, previous research suggested conducting regular exercises according to defined accident scenarios might be useful in providing preparedness among people at the time of incidents [10, 11]. The occurrence of tragic accidents in Iranian universities revealed that they have been slow to apply safety and health principles to the management of risks within processes [3, 4, 12, 13].

Current literature on work health and safety indicated that university institutions need to develop policies and programs to identify, measure, evaluate, and reduce work-related risks to maintain a safety and health environment in their settings [14]. An understanding of what can happen in the format of various scenarios based on identified hazards will enable authorities to invest in providing resource and to develop plans and procedures in keeping people safe and free of danger [15]. Additionally, the literature review highlighted that injuries to people should be the first consideration of the risk assessment. Determining the vulnerability of other at-risk assets such as buildings, equipment, utility systems, and raw materials from hazards would be considered in the next stages [16].

Although universities routinely conduct training programs related to HSE, there is no detailed information concerning the type of hazards that exist in the university environment. Therefore, a risk reduction program must be based on a comprehensive risk assessment. To address this gap, we developed and performed a detailed risk assessment, which can then be addressed in training and risk reduction activities. This study aimed to develop a systematic approach of risk assessment to predict HSE incidents and related injuries in academic settings. Besides providing the required documentation concerning the ranking of deterministic risks and proposing the risk reduction action plan, the authorities of campus would be able to request for funds to remove risky incidents and events in academic settings.

## Methods

### Study design

A cross-sectional study was conducted at School of Public Health of Semnan University of Medical Sciences (SUMS) from June through July 2018. A risk assessment method was developed to evaluate HSE hazards for the indoor and outdoor environment.

### Study instruments and implementation

In the first step, an on-site survey checklist was developed based on the “Hazard Vulnerability Assessment” proposed by University of California and “Aberystwyth University General Risk Assessment Checklist”. These instruments have been applied in previous studies to determine HSE hazards [17]. Additionally, procedures included a literature review, observation, and interview to examine potential hazards. We listed risks through a detailed and comprehensive review of the literature concerning recent accidents or incidents within universities. Our research team conducted an on-site survey to collect details of potential HSE hazards and the possible impacts on the academic settings. Overall, the developed checklist consisted of three sections. Sections A, B, and C were used to identify the types of HSE hazards, respectively. Sections A and B consisted of 13 items, and section C consisted of five items to recognize the relevant hazards in academic settings (Additional file 1). Three trained students performed the survey and completed the checklist, and identified hazards in the determined locations.

In the second step, a team of experts quantified and evaluated the risk levels based on the risk matrix in ISO 31000 [18]. We determined the probable hazardous exposures in terms of staff, students, and visitors. The probability metrics were measured on a five-point scale from “not applicable” to “inevitable” (Table 1). “Not applicable” indicates the incident that will not occur in the next upcoming years. “Doubtful” means the incident that will not likely to occur, “Possible” points to the incident that could occur. “Probable” and “Inevitable” indicate the high probability of incidents that will occur in the future years, respectively.

**Table 1:**
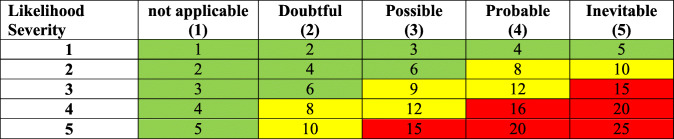
Risk matrix to determine the risk score for each hazard

Finally, the corrective measures were considered in the risk reduction action plan according to the computed risk grading for identified HSE hazards (Additional file 2).

### Risk interpretation^*^

- **1st risk level:** Acceptable risk: 1–3 (Green)
- **2nd risk level:** Corrective measure should be done in the future, if necessary: 3–8 (Green)
- **3rd risk level:** Corrective measure is necessary: 8–13 (Yellow)
- **4th risk level:** As soon as possible corrective measure should be considered: 13–20 (Red)
- **5th risk level:** Stopping the activity and corrective measure should be considered immediately: 20–25 (Red)

^*^Depending on the priority of performing corrective action: 1st and 2nd risk levels were accounted as low risk (Green color), 3rd risk level as moderate risk (Yellow color), 4th and 5th risk levels as high risk in the analysis process (Red color).

Additionally, an integrated approach was used to assess the consequences of hazards. To cover all aspects related to the impacts of identified hazards, the research team determined the severity rate in terms of human, equipment, and institution. To estimate the effect of each identified hazard, we applied two items with a five-point score response. The details of questions and relevant responses for each impact are available in the supplementary file. Responses were scored and averaged to obtain an overall severity score.

### Statistical analysis

Descriptive statistics were applied to measure frequency, percent, and mean of the risks. Analytical tests were used to examine associations among the main variables under study. Kolmogorov-Smirnov test was applied to examine the normal distribution of the variables. Chi-square tests were applied to the qualitative categorical variables including type of hazards, level of risk, and sites to determine the relationship and the significant difference existed between the variables. The Kruskal-Wallis test was used to understand whether type of hazard, measured on an ordinal scale, differed based on risk severity, risk probability, risk score. A correlation test was used to measure the strength of association between two quantitative variables (risk score and total impacts) and the direction of the relationship in this study.

## Results

### Descriptive analysis

**Fig. 1:**
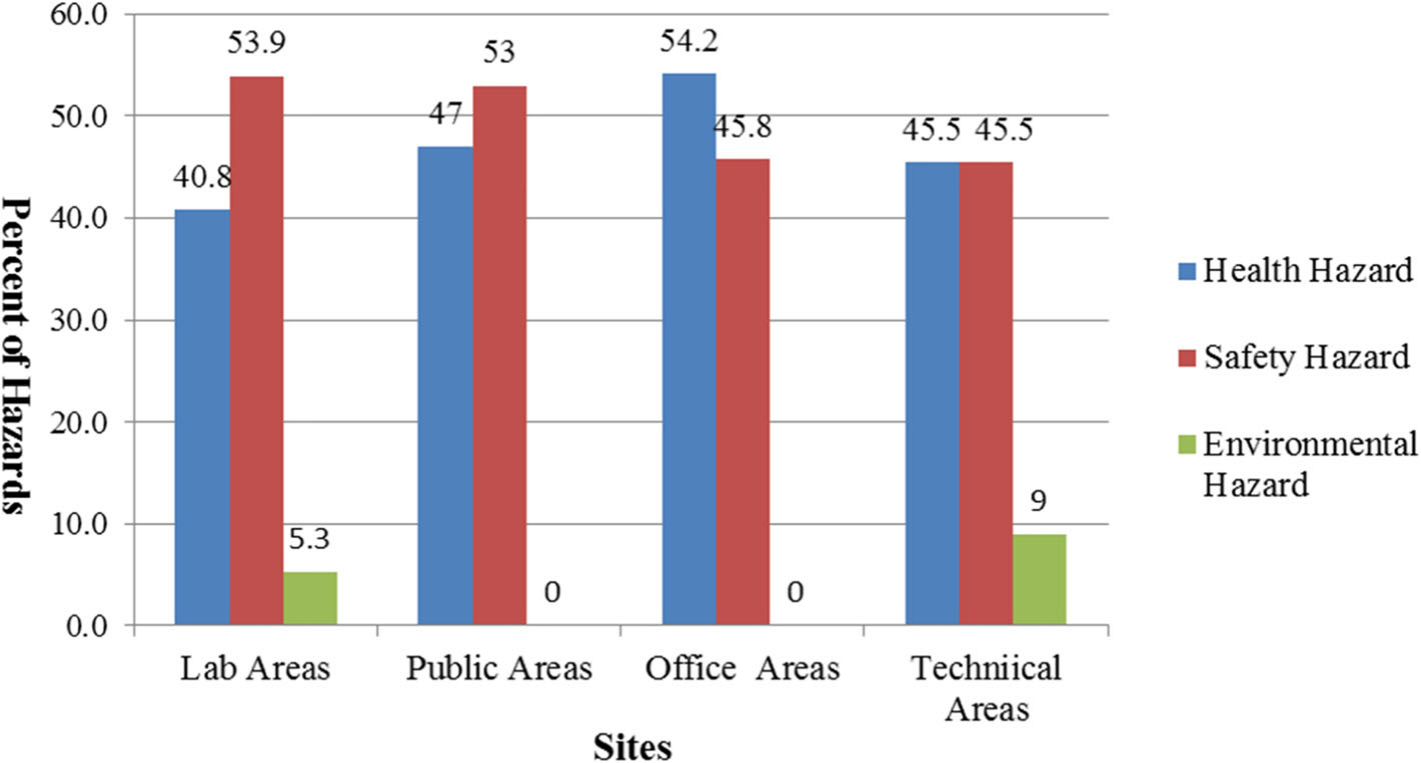
The frequency (%) of identified Health, Safety and Environmental hazards in different locations at academic settings, 2018

Our survey included 38 indoor and outdoor locations in academic settings. Overall, of 297 assessed activities, the frequency and severity of safety hazards were higher in comparison to health and environmental hazards. The total frequency of identified HSE hazards across the university sites were 50.3, 44, and 5.7%, respectively (Fig. 1). Totally, many risks were recognized perceivable to students, faculties, and employees in the surveyed locations. Moreover, we recognized more than half of the potential hazards (55.4%) in public areas and office buildings such as the library, classrooms, computer site, conference hall, faculties’ office, dean office, and administration. Potential hazards associated with serious consequences were allocated to activities housed within chemical and microbiological laboratories and powerhouse facilities. Table 2 demonstrates the details of the hazards in terms of areas within the university and campuses.

**Table 2:**
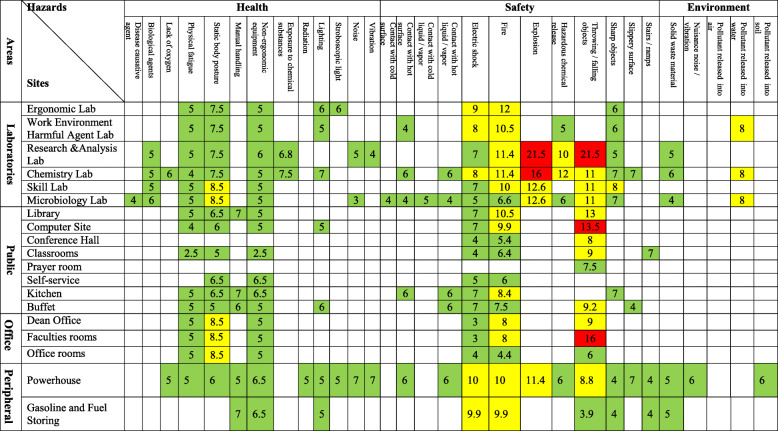
Risk score levels of HSE hazards in understudy sites at the School of Public Health, SUMS, 2018

Our results showed relatively high risks of fire, explosion, electric shock, and hazardous chemical release in the context of classrooms, laboratories, and facilities. Overall, data indicated variations in the probability and severity of risk levels that may be linked to process, practices, infrastructures, and physical structures. The frequency of deterministic first and second risk levels was 74.3%, indicated hazards with a low-risk level. The hazards that allocated to the third risk level were 22.9%, indicated moderate risks. High risks rated at fourth and fifth risk levels consisted of 2.8% of all the identified hazards, required immediate corrective measures.

### Assessment of health hazards

Awkward posture associated, inadequate illumination in office units, and exposure to chemicals in laboratories rated the highest in terms of health hazards, scored 8.5, and 7.5, respectively. Poor work posture was rated 8.5, indicated the highest risk level in terms of frequency and severity due to potential health hazards. Assessment of health hazards showed that the level of risk is related to activities and sites in academic settings. Moreover, in the peripheral facilities located in an enclosed lower ground floor, the leakage of chemicals or fire might result in oxygen deficiency and subsequent health hazards.

### Assessment of safety hazards

Safety-related hazards rated the highest priority in terms of establishing emergency response in the laboratories and public areas. All of the determined hazards at fourth and fifth risk levels were in the domain of safety, which related to the explosion and falling or flying objects with a score of 21.5 (the maximum possible score was 25). This score indicated a significant vulnerability of laboratory facilities. Additionally, the high scores related to the hazardous chemical release, fire, and electrical shock were determined at 12, 11.4, and 10, respectively. Unsafe acts and conditions contributed to such hazards in laboratories and peripheral facilities.

### Assessment of environmental hazards

We measured the hazards due to activities in laboratories and peripheral facilities to the environment. The vulnerability of facilities to effluents was rated 8, indicated the highest risk level of potential hazards to the environment. However, the leakage of chemicals scored a lower level compared to safety and health-related hazards.

### Analytical analyses

Results indicated some relationships among the studied variables, as shown in Tables 3 and 4. The type of hazards and severity of risks were significantly related to sites (*P*-value< 0.01). These significant relations resulted in other similar relationships between the level of risk and sites (*P*-value< 0.05). The finding supports the descriptive results presented in Table 2, indicating the sites and activities might have an impact on the type, severity, and level of risks. Thus, our results supported the alternate hypothesis, stating positive and significant relations between the type, severity, level of risks, and sites in university settings. Accordingly, depending on the nature of the activities, furniture, and materials in the context of academic institutions, the type of hazards, risk severity, and risk level are different.

**Table 3:** Significant results of analytical tests between the variables of the study

Variable	Type of variable	Variable	Type of variable	Value	df	***P***-value
Type of hazard	dependent	Sites	Independent	15.42	6	0.017
Level of risk	dependent	Sites	Independent	21.91	12	0.038
Risk Severity	dependent	Type of hazard	Independent	170.55	2	0.000
Risk Probability	dependent	Type of hazard	Independent	189.06	8	0.000
Risk score	dependent	Total impacts	dependent	0.52	–	0.000

**Table 4:** Comparison of total scores related to HSE hazards in the School of Public Health, SUMS, 2018

Variable	Type of Hazards	Mean Rank	Value	df	***P***-value
Risk Score	Health Hazard	128.15	19.78	2	0.000
	Safety Hazard	170.90			
	Environmental Hazard	113.28			

The correlation ratios with a total risk score computed 0.52, 0.52, and 0.5, showed the effects on individuals, properties, and institutions, respectively. These correlation values showed a significant association between the risk score and categorized impacts (*P*-value < 0.001). The results showed the events had a significant concurrent effect on individuals, properties, and institutions, which could result in an overall higher risk severity score when compared to the impacts of events just limited to the individuals or properties.

Also, the calculated risk score had a significant relation with the type of hazards (*P*-value< 0.001). The estimated mean scores of HSE domains indicated the highest score of 170.9 for safety-related hazards and a statistically higher contribution to the total risk score when compared to health and environmental hazards.

## Discussion

This study introduced a new approach for integrating health, safety and environmental risk factors to have an accurate understanding of the HSE hazards unique to academic settings. Implementing a comprehensive risk assessment provides useful information to plan and develop a risk reduction action plan to promote safety and health in academic Institutions [14, 19, 20].

Our findings demonstrated associations between the type and the level of risks with the assessed locations and facilities. It could be argued that disparities in building structures, geographical places, and work procedures might lead to various risk factors. This is in line with prior study suggested that institutions need to assess their exclusive vulnerabilities and plan their own risk mitigation accordingly [1]. In our study, among HSE hazards, safety-related hazards possessed the highest risk levels in terms of chemical emissions, explosions in laboratories, and falling or flying objects in the indoor locations. Fire, electrical shock, and hazardous chemical release posed serious hazards within safety domains and are more probable to cause an accident and emergency condition in academic settings. Omidvari et al. found similar results in their study at Azad University in Iran, which reported fire risk and accidents in educational buildings, particularly in laboratories [21]. Furthermore, a previous report on the comprehensive management of risks has emphasized on preparing a fire emergency plan for universities and campuses [22].

In the domain of health-related hazards, we assigned the moderate risk level for vulnerabilities to infections in laboratories. Earlier study on academic laboratories reported higher exposure risks of biological hazards [23]. The provision of training courses on health and safety in laboratories particularly for new students at the first of each semester, and designing the suitable layout of safety boxes for syringes and sharp objects will help to decrease infectious risks in laboratories.

In our study, the other health hazard with the moderate risk level was related to awkward and static body posture while performing office work. Prior study confirmed that ergonomic factors might cause musculoskeletal complaints among university staff [24]. Previous action plans such as providing ergonomic chairs and tables, increasing the awareness about ergonomic principles during working activities have shown promising results in decreasing the cumulative fatigue and preventing work-related musculoskeletal disorders [25].

In the domain of environment-related hazards, releasing chemicals into the sewage system can contaminate the underground water with hazardous chemicals. A previous study evaluated a high level of environmental risk related to hazardous chemical effluents from academic laboratories, which might lead to detrimental effect on underground water reservoirs [26, 27]. The provision of a safe disposal is a key element to reduce hazardous impact on environment.

Our study revealed that students, staff, and faculties are generally unaware of how to deal with the physical and chemical hazardous risks they may face in various on campus. The previous study suggested training opportunities related to HSE for students, staff, and faculties which might help increase their awareness and allow them to distinguish what to do during accidents [28, 29]. Thus, an educational institution is recommended to predict HSE education programs including sheltering-in-place or safe evacuation at accidents and emergencies, fire drills, and doing exercises based on the most probable accident scenarios in the proposed risk action plan for all students, staff, and faculties [30, 31]. Furthermore, implementing such these programs will improve the climate of the university environment preparedness in dealing with serious safety, health, and environmental accidents. Previous research emphasized the importance of developing an overall culture of preparedness to ensure the university is properly prepared for all hazards that are unique to its activities during crises. Improving culture and climate through curricula in educational institutions will lead to a cooperative relationship between students and faculties [32].

The result of this risk assessment provided a basis to propose an action plan to evaluate the adequacy of campus prevention and mitigation measures for the most significant campus hazard. Failure in implementing emergency response plans for identified safety hazards will potentially have the capability to generate a crisis. A previous study on the risk assessment for university and college campuses emphasized examining the possible impact of hazards in the institution and planning activities accordingly before, during, and after incidents [33]. Prior studies reported low-cost interventions that might involve reducing major risks and their consequences. Such measures included planning a safe layout, providing the material safety data sheet for using chemicals in the laboratories, and non-structural mitigation measures [19, 34]. Also, it is essential to plan for measures such as maintenance, and regular inspections of fire protection systems, checking the earth systems of electrical equipment and assessment of structural and non-structural safety in the proposed risk reduction action plan. Importantly, attracting the participation of students, staff, and faculties to develop an action plan could increase the sensitivity of individuals for implementing it. A study on risk assessment management in Italy reported the benefits of a supervising sector to provide HSE rules and standards for higher education institutions [11]. Also, another report in Chile emphasized on the supervision of the implementation of these risk-reduction programs [35].

## Conclusion

This study indicated that comprehensive HSE risk assessment provides the basis for establishing a framework for an action plan against incidents. Taking proactive behavior by improving ties among students, staff, and faculties will increase risk-reduction preparedness in the context of university settings. Our study revealed a comprehensive and detailed assessment of the risks to the HSE across all university activities that could help to identify minor and major risks in developing reasonable practical precautions.

The events such as fire, explosion, and throwing or falling objects have a higher risk of occurring and were identified to cause damage and injuries in various areas of the institution. Based on our risk assessment, a chemical safety management plan should be implemented as soon as possible for the risks of fire and explosion in laboratory activities, which scored the highest risk in this study. Additionally, non-structural mitigation measures should be provided as soon as possible in laboratories and general spaces of academic settings based on achieved risk score to hazard of throwing or falling objects. Meanwhile, health and safety education programs increase the awareness of people that has an essential role in the risk understanding and providing the safety climate in university. Furthermore, relevant exercises based on determined hazards are necessary to strengthen preparedness in case of critical situations. Attracting individual participation in developing risk reduction measures encourages students, staff, and faculties to take preparedness seriously.

Our study suggests the application of the developed comprehensive risk assessment method in other schools and academic settings to compare the generalizability of this method for assessing HSE hazards before developing a risk reduction action plan.

## Supplementary information

**Additional file 1.** Hazards Checklist.

**Additional file 2.** Risk Assessment Checklist.

## Data Availability

The data supporting the conclusions in this article are available in the additional files. Data supporting study findings are available upon request.

## Abbreviation

HSE: Health, Safety, Environment
