# Supplementary material for: Applying health, safety, and environmental risk assessment at academic settings
Source: BMC Public Health. 2020 Sep 1;20:1328. doi: 10.1186/s12889-020-09419-5 (PMC7466792; doi:10.1186/s12889-020-09419-5)
Supplement: Supplementary file 1 — Additional file 1. Hazards Checklist. [file 12889_2020_9419_MOESM1_ESM.docx]

**Hazards Checklist**

**Place: …………………………….. Assessor: ………………….**

| **Hazard type** | | | | | | | | |
| --- | --- | --- | --- | --- | --- | --- | --- | --- |
| **Health** | | | **Safety** | | | **Environment** | | |
| Noise | Yes | No |  | Yes | No |  | Yes | No |
| Vibration |  |  | Stairs / ramps |  |  | Solid waste material |  |  |
| Lighting |  |  | Slippery surface |  |  | Nuisance noise / vibration |  |  |
| Stroboscopic effect |  |  | Sharp objects |  |  | Pollutant released into air |  |  |
| Radiation |  |  | Throwing / falling objects |  |  | Pollutant released into water |  |  |
| Exposure to chemical substances |  |  | Hazardous chemical release |  |  | Pollutant released into soil |  |  |
| Non-ergonomic equipment |  |  | Explosive material |  |  |  |  |  |
| Manual handling |  |  | Flammable material |  |  |  |  |  |
| Static body posture |  |  | Electric shock |  |  |  |  |  |
| Physical fatigue |  |  | Contact with hot liquid / vapor |  |  |  |  |  |
| Lack of oxygen |  |  | Contact with cold liquid / vapor |  |  |  |  |  |
| Biological agents |  |  | Contact with hot surface |  |  |  |  |  |
| Disease causative agent |  |  | Contact with cold surface |  |  |  |  |  |
