# Supplementary material for: Applying health, safety, and environmental risk assessment at academic settings
Source: BMC Public Health. 2020 Sep 1;20:1328. doi: 10.1186/s12889-020-09419-5 (PMC7466792; doi:10.1186/s12889-020-09419-5)
Supplement: Supplementary file 2 — Additional file 2. Risk Assessment Checklist. [file 12889_2020_9419_MOESM2_ESM.docx]

**Risk Assessment Checklist**

**Place: ………………………. Assessor: ……………………….**

| No. | Hazard Type | Persons At risk | Risk probability | Risk severity | | | Risk severity mean | Control measures required |
| --- | --- | --- | --- | --- | --- | --- | --- | --- |
|  |  |  |  | human | Structure & equipment | Institutional  services |  |  |
| 1 |  |  |  |  |  |  |  |  |
| 2 |  |  |  |  |  |  |  |  |
| 3 |  |  |  |  |  |  |  |  |
| 4 |  |  |  |  |  |  |  |  |
| 5 |  |  |  |  |  |  |  |  |
